# Temporal increase of platelet mitochondrial respiration is negatively associated with clinical outcome in patients with sepsis

**DOI:** 10.1186/cc9337

**Published:** 2010-11-24

**Authors:** Fredrik Sjövall, Saori Morota, Magnus J Hansson, Hans Friberg, Erich Gnaiger, Eskil Elmér

**Affiliations:** 1 Mitochondrial Pathophysiology Unit, Laboratory for Experimental Brain Research, Department of Clinical Sciences, Lund University, Sölvegatan 17, SE-221 84, Lund, Sweden; 2 Intensive Care Unit 4131, Copenhagen University Hospital, Rigshospitalet, Blegdamsvej 9, DK-2100, Copenhagen, Denmark; 3 Department of Clinical Physiology, Skåne University Hospital, Getingevägen 4, SE-221 85, Lund, Sweden; 4 Department of Emergency Medicine, Skåne University Hospital, Getingevägen 4, SE-221 85, Lund, Sweden; 5 Department of Visceral, Transplant and Thoracic Surgery, D. Swarovski Research Laboratory, Innrain 66/6, A-6020, Innsbruck Medical University, Innsbruck, Austria; 6 Department of Clinical Neurophysiology, Skåne University Hospital, Getingevägen 4, SE-221 85, Lund, Sweden

## Abstract

**Introduction:**

Mitochondrial dysfunction has been suggested as a contributing factor to the pathogenesis of sepsis-induced multiple organ failure. Also, restoration of mitochondrial function, known as mitochondrial biogenesis, has been implicated as a key factor for the recovery of organ function in patients with sepsis. Here we investigated temporal changes in platelet mitochondrial respiratory function in patients with sepsis during the first week after disease onset.

**Methods:**

Platelets were isolated from blood samples taken from 18 patients with severe sepsis or septic shock within 48 hours of their admission to the intensive care unit. Subsequent samples were taken on Day 3 to 4 and Day 6 to 7. Eighteen healthy blood donors served as controls. Platelet mitochondrial function was analyzed by high-resolution respirometry. Endogenous respiration of viable, intact platelets suspended in their own plasma or phosphate-buffered saline (PBS) glucose was determined. Further, in order to investigate the role of different dehydrogenases and respiratory complexes as well as to evaluate maximal respiratory activity of the mitochondria, platelets were permeabilized and stimulated with complex-specific substrates and inhibitors.

**Results:**

Platelets suspended in their own septic plasma exhibited increased basal non-phosphorylating respiration (state 4) compared to controls and to platelets suspended in PBS glucose. In parallel, there was a substantial increase in respiratory capacity of the electron transfer system from Day 1 to 2 to Day 6 to 7 as well as compared to controls in both intact and permeabilized platelets oxidizing Complex I and/or II-linked substrates. No inhibition of respiratory complexes was detected in septic patients compared to controls. Non-survivors, at 90 days, had a more elevated respiratory capacity at Day 6 to 7 as compared to survivors. Cytochrome *c *increased over the time interval studied but no change in mitochondrial DNA was detected.

**Conclusions:**

The results indicate the presence of a soluble plasma factor in the initial stage of sepsis inducing uncoupling of platelet mitochondria without inhibition of the electron transfer system. The mitochondrial uncoupling was paralleled by a gradual and substantial increase in respiratory capacity. This may reflect a compensatory response to severe sepsis or septic shock, that was most pronounced in non-survivors, likely correlating to the severity of the septic insult.

## Introduction

Multiple organ failure (MOF) is the leading cause of death in patients with severe sepsis and septic shock [1]. The cause of MOF is largely unexplained and the pathogenesis is likely complex. Since the failing organs do not undergo necrosis or apoptosis to any large extent [2], there is a possibility of full recovery with supportive treatment.

Evidence that mitochondrial alterations contribute to the pathogenesis of MOF has been gathered in animal as well as human studies (reviewed in [3]) although differences exist depending on the tissue studied [4]. Restoration of mitochondrial function has also been suggested as a prerequisite in recovery from MOF. Initial depletion of mitochondrial DNA (mtDNA) and subsequent activation of mitochondrial biogenic factors and restoration of mtDNA copy number were seen in a murine model of sepsis [5] and recently, increased transcripts of mitochondrial biogenetic markers was associated with survival in patients with severe sepsis and septic shock [6].

Mitochondria are unique in that they contain their own genome that is maternally inherited. Compared to nuclear DNA, mtDNA is more prone to damage because the DNA is not bound to histones and has reduced capacity of DNA repair [7]. A fall in mtDNA in mononuclear cells was recently shown in patients with sepsis [8] and a temporal increase of mtDNA in blood cells from septic patients has been associated with improved outcome [9].

Platelets are anucleated cells that contain two to eight mitochondria per cell [10] with their main function in the process of coagulation. Recently, they have been shown to play a role in innate immunity, containing toll-like receptors and interplaying with other immune cells [11]. Further, platelet mitochondria have been proposed to serve as a marker for changes in mitochondrial function occurring in senescence and age-related diseases [12-14].

Although mitochondrial biogenesis seems to be triggered early on in sepsis the temporal evolution or functional outcome has not been avidly studied. This is in part due to the ethical and practical problems of obtaining adequate mitochondrial samples from vital organs in critically ill patients. In order to address this question we examined changes in platelet mitochondrial respiratory function during the first week in patients with severe sepsis or septic shock and evaluated how these changes correlated with clinical parameters, severity scores and mortality. Using high-resolution respirometry we analyzed integrated mitochondrial function in both intact platelets with preserved intra- and extracellular environment as well as the contribution of individual respiratory complexes in permeabilized cells. By evaluating platelet mitochondrial function in the presence or absence of the patients' own plasma we addressed the possible influence of soluble factors affecting respiratory capacity.

Preliminary data of this study have been presented at the annual International Sepsis Forum meeting 2010 [15].

## Materials and methods

### Study population

This study was approved by the scientific ethical committee of Copenhagen County, Denmark (H-C-2008-023) and the regional ethical review board of Lund, Sweden (113/2008). Patients were recruited from the intensive care units (ICU) of Lund University Hospital and Copenhagen University Hospital, Rigshospitalet. Written, informed consent was obtained from the patient or next of kin. In Denmark, consent from the patient's primary health care physician was also required if the patient was unconscious. Eighteen patients with severe sepsis or septic shock, as previously defined [16], were included within 48 h after their admission to the ICU. Diagnosis of sepsis should have been made no more than 24 h prior to ICU admission. Patients with platelet count <10 × 10^9^/L, pregnancy, known mitochondrial disease or hematological malignancy were excluded. Blood samples were taken at three different time points during the first week following admission to the ICU; within the first 48 h (Day 1 to 2), on Day 3 to 4 and Day 6 to 7. If a patient received platelet transfusion a minimum of six hours had to pass before blood sampling. Eighteen healthy blood donors served as controls following written, informed consent.

### Chemicals and sample preparation

All chemicals were purchased from Sigma-Aldrich (St Louis, MO, USA) if not stated otherwise. In patients, a maximal volume of 40 mL of blood was drawn from an existing arterial line in K_2_EDTA tubes (Vacuette^®^, Greiner Bio-One GmbH, Kremmünster, Austria). In controls, blood samples were taken at the time of planned donation via venous puncture in K_2_EDTA tubes. Platelets were freshly prepared by centrifugation 10 to 15 minutes at 300 × *g *resulting in a platelet-rich plasma (PRP). This PRP was collected and centrifuged for five minutes at 4,600 × *g *producing a close to cell-free plasma and a platelet pellet. The pellet was resuspended in 1 to 3 mL of plasma by gentle pipeting to obtain a highly enriched PRP.

### High-resolution respirometry

Measurement of mitochondrial respiration was performed in a high-resolution oxygraph (Oxygraph-2k Oroboros Instruments, Innsbruck, Austria [17]) at a constant temperature of 37°C. Platelets were suspended in the 2 mL glass chamber at a concentration of 50 to 200 × 10^6^/mL. Calibration with air-saturated Millipore water was performed daily. For experiments in intact cells, platelets were suspended in either phosphate buffered saline (PBS) with addition of 5 mM glucose or in their own plasma. For respiration measurements in permeabilized cells, platelets were suspended in a mitochondrial respiration medium (MiR05) containing sucrose 110 mM, HEPES 20 mM, taurine 20 mM, K-lactobionate 60 mM, MgCl_2 _3 mM, KH_2_PO_4 _10 mM, EGTA 0.5 mM, BSA 1 g/l, pH 7.1 [17]. Oxygen solubility factors relative to pure water were set to 0.92 for MiR05 and PBS glucose and 0.89 for plasma. Data were collected using software displaying real-time oxygen concentration and oxygen flux, that is, the negative time derivative of oxygen concentration (DatLab software 4.3, Oroboros Instruments, Innsbruck, Austria).

### Experimental protocol for intact cells

Respiration was first allowed to stabilize without any additions at a routine state, that is, in the physiological coupling state controlled by cellular energy demands on oxidative phosphorylation (OXPHOS). Then the ATP synthase inhibitor oligomycin was added to reveal respiration independent of ADP phosphorylation (oligomycin-induced state 4, henceforth denoted as state 4). To evaluate maximal capacity of the electron transfer system (ETS) the protonophore, carbonyl cyanide p-(trifluoromethoxy) phenylhydrazone (FCCP) was titrated until no further increase in respiration was detected. The ETS was then inhibited by adding rotenone (Complex I inhibitor) and antimycin-A (Complex III inhibitor). The remaining, primarily non-mitochondrial oxygen consumption (residual) was subtracted from the different respiratory states in further analyses. In intact cells, to determine the relative contribution of the different respiratory states, a control ratio was calculated as the ratio of maximal FCCP-stimulated respiration and state 4 respiration.

### Experimental protocol for permeabilized cells

The next protocol was established to separate the respiratory capacities through Complex I and Complex II as achieved in conventional respirometric protocols. In addition, maximal phosphorylating and non-phosphorylating respiration were measured as stimulated by combined succinate plus NADH-related substrate supply. This substrate combination is required as a basis to reconstitute the citric acid cycle function in permeabilized cells or isolated mitochondria, with convergent Complex I and II electron input [18]. Sequential additions were performed in a substrate, uncoupler, inhibitor titration (SUIT) protocol. Platelets were allowed to stabilize at routine respiration without exogenous substrates in MiR05, and were then permeabilized with digitonin in order to access the mitochondria with the different respiratory substrates and ADP. In a different set of experiments the optimal concentration of digitonin was set to 1 μg/1 × 10^6 ^platelets (data not shown). Respiration through Complex I, driven by NADH-related substrates, was evaluated by adding first malate (5 mM) and pyruvate (5 mM), then ADP (1 mM), and finally glutamate (5 mM) (CI_OXPHOS_, or state 3_CI_). Maximal OXPHOS capacity by convergent input through both Complex I and Complex II was obtained by sequentially adding 10 mM succinate (CI+II_OXPHOS_, or state 3_CI+II_) after NADH-related substrates and ADP. State 4 (with CI and CII substrates present) was evaluated by adding oligomycin and maximal capacity of the ETS was obtained by titrating FCCP (CI+II_ETS_). Inhibition of Complex I by rotenone revealed the ETS capacity supported by succinate through Complex II alone (CII_ETS_). Finally, residual oxygen consumption was determined by addition of antimycin-A. In permeabilized cells, control ratios were calculated for both maximal capacity of OXPHOS and ETS by dividing the respective rate with state 4 respiration. After experiments, analyzed samples were stored at -80 °C.

### Determination of platelet mtDNA content

The analysis of platelet mtDNA content was adapted from [19] with modifications. Frozen samples were thawed and diluted 500 times in a lysis buffer (10 mM TRIS-HCl, 1 mM EDTA, salmon sperm DNA 1 ng/μl, pH 8.0). 10 μl of this dilution was amplified in a 25 μl PCR reaction containing 1 × Power SYBR^® ^Green PCR Master Mix using an ABI Prism 7000 real-time PCR machine (Applied Biosystems Inc., Foster City, CA, USA) and 100 nM of each primer (Eurofins MWG-operon, GmbH, Ebersberg, Germany). The primers targeted the human mitochondrial COX-1 gene (forward: CCC CTG CCA TAA CCC AAT ACC A, reverse: CCA GCA GCT AGG ACT GGG AGA GA). The threshold cycle (C_t_) values were related to a standard curve using cloned PCR products (kindly provided by P. Schjerling University of Copenhagen, Denmark). Due to relatively high variation, samples were analyzed in pentaplicate.

### Cytochrome *c *determination

Human cytochrome *c *(Cyt *c*) content was quantified using an immunoassay kit (DCTC0, Quantikine^®^, R&D systems, Abingdon, UK). Frozen samples where thawed and sonicated and subsequently processed according to the manufacturer's instructions.

### Data analysis

**Figure 1:**
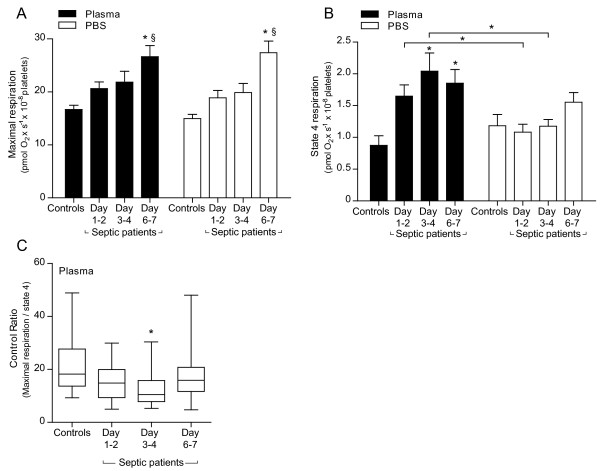
**Mitochondrial respiration of intact platelets suspended in their own plasma or PBS glucose**. **A**. Maximal respiration induced by titration of the protonophore FCCP demonstrated a significant increase in both media, from Day 1 to 2 to Day 6 to 7 as well as compared to controls. **B**. State 4 respiration in presence of the ATP synthase inhibitor oligomycin was significantly higher on Day 3 to 4 and Day 6 to 7 in platelets suspended in plasma compared to controls, whereas no difference was seen in PBS glucose. **C**. The control ratio (maximal respiration/state 4 respiration) in platelets incubated in patients' own plasma was significantly lower at Day 3 to 4 compared to controls. Mean values ± SEM (A, B) and median with interquartile range and range (C), *n *= 17 to 18, *P < 0.05.

All absolute values are presented as mean ± SEM or individual values. Ratios are presented as median with range. Graph Pad PRISM (GraphPad Software version 5.01, La Jolla, CA, USA) was used for statistical evaluation. Analysis between two groups was performed using unpaired or paired Student's *t*-test as appropriate. For comparison of three or more groups one-way ANOVA or repeated measurements ANOVA were used as appropriate. Kruskal-Wallis or Friedmans non-parametric tests were used for comparisons of ratios and Mann-Whitney *U *test for mortality data. For missing values in repeated measurements (in total two values in separate patients) "last value carried forward" was employed. One negative value in state 4 respiration was omitted in the analysis presented in Figure 1B. A *P*-value less than 0.05 was considered significant.

## Results

### Study population

A total of 18 patients with severe sepsis or septic shock were studied and 18 healthy blood donors served as controls. Table 1 shows demographics, source of sepsis, severity of illness and outcome of the septic patients and demographics for the healthy controls. Table 2 shows clinical characteristics of the septic patients during the first week at the ICU. A total of four patients received platelet transfusion; two at the day of sample one and two at the day of sample three. No medication with known platelet interaction was administered during the study.

**Table 1:** Demographic and clinical characteristics of patients and controls

	Patients (*n *= 18)	Controls (*n *= 18)
Age	64 (50 to 73)	51 (38 to 60)
Male/Female	14/4	12/6
Source of sepsis:		
- Chest	8	
- Abdominal	7	
- Soft tissue	3	
SAPS II	46 (41 to 51)	
APACHE II	24 (16 to 26)	
Severe sepsis/septic shock	3/15	
90-day outcome dead/alive	6/12	

Data presented as median with interquartile range (IQR) or number of patients. SAPS, simplified acute physiology score; APACHE, acute physiology and chronic health evaluation score.

**Table 2:** Clinical characteristics of patients at the time of blood sampling

	Day of sample 1	Day of sample 2	Day of sample 3
SOFA (median (IQR))	10 (7 to 13)	9 (5 to 11)	7 (3 to 9)
Noradrenaline infusion rate (μg/kg/min)	0.19 ± 0.04	0.09 ± 0.04	0.01 ± 0.01
Maximum lactate (mmol/L)	3.5 ± 0.6	2.0 ± 0.3	1.3 ± 0.3
Average B-glucose level (mmol/L)	9.5 ± 0.4	8.6 ± 0.6	8.0 ± 0.4
Platelet count (x 10^9^/L)	153 ± 24	146 ± 23	146 ± 22
CRP (mg/L)	203 ± 21	131 ± 24	57 ± 16
PCT (ng/mL)	73 ± 51	30 ± 21	4 ± 2
Platelet transfusion (patients)	2	0	2
Dialysis (patients)	3	3	3

CRP, C-reactive protein; IQR, interquartile range; PCT, procalcitonin; SOFA, sequential organ failure assessment.

### Mitochondrial respiration of intact platelets

In intact platelets, FCCP-titrated maximal respiration gradually increased in septic patients during the first week after admission to the ICU. The maximal respiration (pmol O_2 _x s^-1 ^× 10^-8 ^platelets) increased significantly from Day 1 to 2 to Day 6 to 7, 29% in plasma (20.6 ± 1.2 vs 26.7 ± 2.1) and 45% in PBS glucose (18.9 ± 1.4 vs 27.4 ± 2.2). Compared to controls the increase was 60% in plasma (16.7 ± 0.8 vs 26.7 ± 2.1) and 85% in PBS glucose (15 ± 0.8 vs 27.4 ± 2.2) at Day 6 to 7 (Figure 1A). State 4 respiration in PBS glucose was not different from controls and did not change significantly over the week. In contrast, state 4 respiration determined in the patients' own septic plasma was significantly higher compared to controls at Day 3 to 4 and Day 6 to 7 and also significantly higher compared to PBS glucose at Day 1 to 2 and Day 3 to 4 but not at Day 6 to 7 (Figure 1B). When adjusted for Cyt *c *content, state 4 respiration in plasma was significantly higher, compared to control, also at Day 1 to 2 (data not shown). The control ratio (FCCP-titrated maximal respiration/state 4 respiration) of mitochondria in septic plasma decreased over the first days, due to the increase in state 4 respiration, and was significantly lower than controls at Day 3 to 4 (Figure 1C).

### Mitochondrial respiration of permeabilized platelets

**Figure 2:**
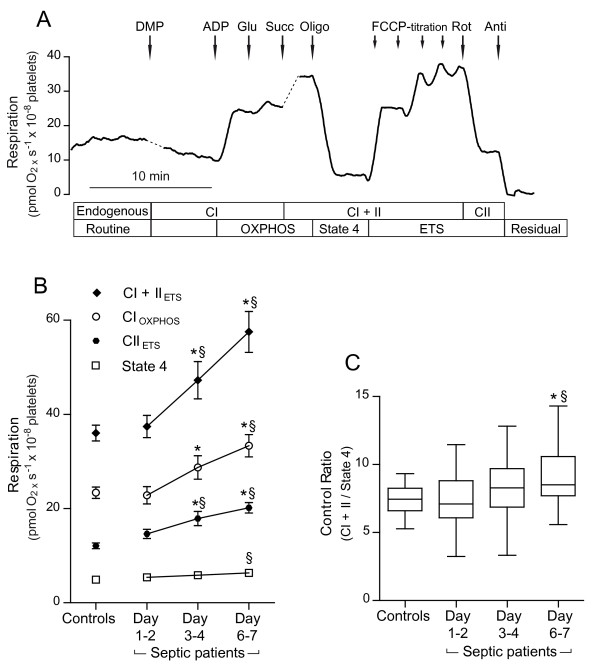
**Mitochondrial respiration of permeabilized platelets**. **A**. Representative trace of oxygen consumption rate using a substrate, uncoupler, inhibitor titration protocol. Respiratory complexes activated and the induced respiratory states are defined below the x-axis. Platelets were permeabilized with digitonin with simultaneous addition of malate and pyruvate (DMP). Oxidative phosphorylation (OXPHOS) was stimulated by subsequent addition of ADP followed by an additional Complex I (CI) substrate glutamate (Glu). Addition of the Complex II (CII)-linked substrate succinate (Succ) enabled convergent electron input via both complex I and complex II. OXPHOS was inhibited by oligomycin (Oligo) revealing state 4 respiration. Maximal respiratory capacity of the electron transfer system (ETS) was induced by titration of FCCP. Inhibition of Complex I by rotenone (Rot) revealed Complex II-supported respiration. The Complex III inhibitor antimycin-A (Anti) left residual, primarily non-mitochondrial oxygen consumption. **B**. Temporal changes of different respiratory states during the first week of sepsis in patients compared to controls. Complex I-dependent respiration during oxidative phosphorylation (CI_OXPHOS_), FCCP-titrated maximal respiration with complex II (CII_ETS_), and convergent substrate input (CI + II_ETS_) all increased significantly in patients during the first week of sepsis both compared to Day 1 to 2 and to controls. State 4 was only increased at Day 6 to 7 compared to controls. Of note, there was no significant difference in respiratory capacity of any respiratory state in patients at the earliest time point analyzed, Day 1 to 2 of admission, compared to controls. **C**. The control ratio (CI + II_ETS_/state 4) increased significantly during the first week of sepsis both compared to Day 1 to 2 as well as to controls. Mean values ± SEM (B) and median ± range (C), *n *= 16 to 18, *P < 0.05 compared to Day 1 to 2, § *P *< 0.05 compared to controls.

A representative trace of the SUIT protocol used in permeabilized platelets is depicted in Figure 2A. In the presence of saturating Complex I substrates respiration, CI_OXPHOS_, increased by 47% from Day 1 to 2 to Day 6 to 7 and was 43% higher Day 6 to 7 compared to controls (22.7 ± 1.8, 33.3 ± 2.4 and 23.4 ± 1.2, respectively) (Figure 2B). No differences in the relative contribution of the different Complex I-linked respiratory substrates (pyruvate, malate, glutamate) were detected between controls and septic patients (data not shown). CII_ETS _increased by 39% from Day 1 to 2 to Day 6 to 7 and was 67% higher Day 6 to 7 compared to controls (14.6 ± 1.0, 20.2 ± 1.1 and 12.1 ± 0.7, respectively). CI + II_ETS _increased by 54% from Day 1 to 2 to Day 6 to 7 and was 60% higher Day 6 to 7 compared to controls (37.3 ± 2.4, 57.5 ± 4.3 and 36.0 ± 1.7, respectively). State 4 respiration also increased gradually to some extent in septic patients and was significantly higher compared to controls at Day 6 to 7 with a difference of 29% (4.9 ± 0.2 vs. 6.3 ± 0.4) (Figure 2B). Control ratios for ETS (CI + II_ETS _/state 4) and OXPHOS (CI + II_OXPHOS _/state 4) both increased significantly during the first week of sepsis due to the more pronounced increase in maximal respiratory capacity compared to the increase in state 4 respiration (Figure 2C). No significant changes in respiration rates of permeabilized platelets were detected at Day 1 to 2 compared to controls.

### Respiratory changes in platelet mitochondria in relation to clinical parameters and mortality

**Figure 3:**
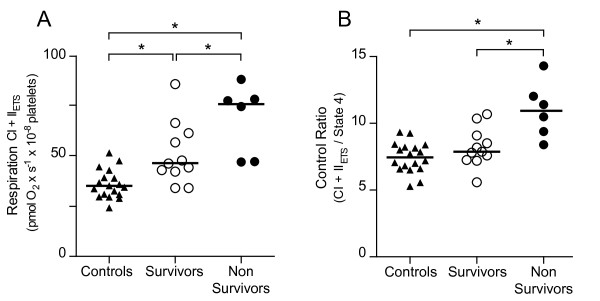
**Platelet mitochondrial respiration related to three-month mortality**. **A**. Maximal FCCP-titrated respiration (CI + II_ETS_) and **B**. The control ratio (CI + II_ETS _/state 4) at Day 6 to 7 in survivors and non-survivors at three months following sepsis versus controls. Non-survivors demonstrated both a higher maximal respiration value and a higher corresponding control ratio than survivors. Individual values and medians are shown. *P < 0.05.

Patients were divided into survivors and non-survivors according to 90-day mortality that was 33% (6/18). At Day 6 to7 both FCCP-induced maximal respiration (CI + II_ETS_) as well as the corresponding control ratio was significantly higher in non-survivors compared to survivors (Figure 3). A significant difference in non-survivors compared to survivors was also seen in CI_OXPHOS _as well as the control ratio (CI + II_OXPHOS_/state 4) with a similar trend in CI + II_OXPHOS _and CII_ETS _respiration states (data not shown). We did not find any correlation between mitochondrial respiration and severity of illness as measured by APACHE II, SAPS- and SOFA score and noradrenaline requirement at any of the measured time points (data not shown).

### Quantification of platelet mtDNA and Cyt *c *content

**Figure 4:**
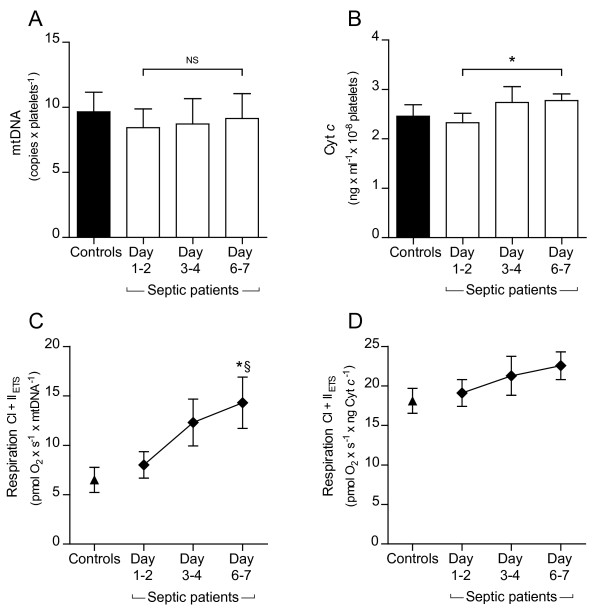
**Quantification of mitochondrial DNA (mtDNA) and cytochrome c (Cyt *c*) content in platelets**. **A**. Platelet mtDNA content did not change in the septic patients during the first week or compared to controls. **B**. Cyt *c *content increased significantly from Day 1 to 2 to Day 6 to 7 in septic patients but not compared to controls. **C**. Maximal FCCP-titrated respiration (CI + II_ETS_) adjusted to mtDNA increased similarly as when adjusted to platelet count. **D**. Maximal respiration (CI + II_ETS_) adjusted to Cyt *c *demonstrated an increasing trend but no significant change. Mean values ± SEM, *n *= 16-18, *P < 0.05 compared to Day 1 to 2, § *P *< 0.05 compared to controls.

In order to determine changes of mitochondrial number and protein content in analyzed platelets, we measured two different parameters, mtDNA and Cyt *c*. The amount of mtDNA did not differ in platelets of septic patients compared to controls and did not change during the course of sepsis (Figure 4A). In contrast, we found a significant increase in mitochondrial Cyt *c *content in platelets of septic patients from Day 1 to 2 to Day 6 to 7 but not compared to controls (Figure 4B). Respiration parameters adjusted to mtDNA content still showed a significant increase over the week in septic patients (example given in Figure 4C). In both intact as well as permeabilized cells, respiration parameters adjusted to Cyt *c *did not change significantly over the studied time period (example given in Figure 4D).

## Discussion

In this study we demonstrate several alterations in platelet mitochondrial respiratory function during the course of the first week in patients admitted to the ICU due to severe sepsis or septic shock. First, plasma from septic patients induced an elevated state 4 respiration (uncoupling) resulting in a decreased control ratio (FCCP-titrated maximal respiration/state 4 respiration) which was not seen when plasma was removed and platelets were incubated in "clear" respiration media such as PBS or the mitochondrial respiration medium MiR05. Second, mitochondrial respiratory capacity increased gradually and extensively during the week which was paralleled by an increase in mitochondrial Cyt *c *content. Third, non-survivors had a significantly more elevated level of respiratory capacity at Day 6 to 7 compared to survivors.

Platelets incubated in their own septic plasma had a significantly elevated state 4 respiration at Day 3 to 4 and Day 6 to 7 compared to controls resulting in a decreased control ratio. Increased state 4 respiration and reduced control ratios have been demonstrated previously in sepsis. D'Avila *et al*. found mitochondrial uncoupling in brain homogenates in a 24 h cecal ligation and puncture model (CLP) model in mice [20]. Also, plasma taken from septic patients at Day 1 and Day 7 induced increased state 4 respiration in peripheral blood mononuclear cells from healthy controls [21]. State 4 respiration, that is, when oxidation of respiratory substrates is not coupled to ATP synthesis, is the result of "leak" of protons, slip of protons within the proton pumps and exchange of cations across the inner mitochondrial membrane. The formation of reactive oxygen species (ROS) could also contribute to some extent to oxygen consumption and in the case of whole and permeabilized cell analysis, also non-mitochondrial oxidative processes. However, both of these latter processes were removed from the respiratory rates in the present study by subtracting the low residual oxygen consumption following Complex III inhibition. The mitochondrial permeability transition (mPT) has been implicated in sepsis [22,23] but cannot readily explain the elevated state 4 respiration in the present data. Activation of mPT leads to loss of mitochondrial matrix substrates as well as dissipation of the proton-motive force which uncouples as well as inhibits respiration [24], but no inhibition of the ETS was detected in the present investigation. Uncoupling proteins (UCPs) have been implicated in sepsis where UCP3 has been shown to be upregulated in muscle in a CLP model in rats and UCP2 deficient mice were protected from LPS-induced liver failure [25,26]. Also, ROS production is increased in sepsis and a proton leak; ROS feedback loop has been suggested where ROS increase proton leak which in turn reduces ROS production via lowering of the proton-motive force [27,28]. In the present study, no increase in state 4 respiration was found when plasma was removed and platelets were incubated in PBS glucose. This suggests presence of a soluble or a rapidly metabolized factor in septic plasma that can induce mitochondrial uncoupling either alone or as an activator of endogenous pathways such as uncoupling proteins. Whatever the cause of the uncoupling it remains to be elucidated if this stands for a true pathophysiological mechanism or constitutes a protective mechanism in regulating ROS production.

Concomitantly, we found that the respiratory capacity of the platelet mitochondria increased by 29 to 54% depending on experimental conditions and up to about 85% compared to controls. The temporal increase was seen both at the level of individual complexes as well as in integrated respiration of intact platelets and was significant already early in the disease process (Day 3 to 4). mtDNA copy number per platelet remained stable over the seven days investigated suggesting that the increase did not come from an increased number of organelles in the cell and is in accordance with a previous study [9]. In contrast, we noted a 19% rise in Cyt *c *protein, used here as a marker of cellular content of mitochondrial proteins. The body copes with increasing demands of energy supply via mitochondrial biogenesis [29]. Cytokines which are known to be elevated in sepsis have also been shown to activate regulators of mitochondrial biogenesis such as peroxisome profilerator-activated receptor γ coactivator -1α (PGC-1α) [30]. Biogenesis has also been proposed to play an important part in the recovery following sepsis. In a murine model of *Staphylococcus aureus *sepsis, Haden *et al*. demonstrated an early fall (Day 1) in liver mtDNA copy number. Subsequently they noted an increase in transcription of nuclear respiratory factor (NFR)-1, NRF-2, mitochondrial transcription factor A (Tfam) and PGC-1α already at Day 2 after the induction of sepsis. At Day 3 mtDNA copy numbers were restored to normal values [5]. Apart from increased number of organelles, biogenesis can also lead to increased density of respiratory complexes per organelle [31,32] and reversible protein phosphorylation has been suggested to be a key mechanism in modulating the post-translational function of respiratory complexes [33]. Some of the observed increase in respiratory capacity could be related to a high turnover of platelets in sepsis creating a more freshly produced pool of mitochondria being studied. The difference in respiratory capacity, if any, of newly produced platelets compared to the circulating pool is not known. However, we find it unlikely that such a difference may explain the pronounced increase in respiration rate, as much as 85%, found in the septic patients. Also, there was no correlation between platelet count or change in platelet count and respiratory capacity at any of the time points studied. Four patients received platelet transfusions on the day of sampling which could confound the results. However, excluding the patients' data completely or from specific days of transfusion did not influence the main findings or conclusions of the study (data not shown). The results from the present study support the hypothesis that in the recovery from sepsis with organ failure there is increasing metabolic demands that is met via a progressive rise in mitochondrial respiratory capacity. The temporal increase in Cyt *c *suggests that this process is mediated, at least in part, via a higher mitochondrial density of ETS-related proteins. At present the correlation between a given increase in ETS proteins and the resulting change in respiratory capacity is unknown.

Surprisingly, non-survivors at 90 days displayed significantly elevated levels of respiratory capacity compared to survivors at Day 6 to 7; both in absolute values as well as expressed as control ratios. This stands somewhat in contrast with recent findings that survival after severe sepsis was associated with early activation of mitochondrial biogenesis [6]. Also, survivors from septic or cardiogenic shock showed an increase in mtDNA/nDNA ratio in blood cells compared to non-survivors [9] although this finding could have been caused by a variation of different leucocyte proportions [8]. Tissue differences set aside, the findings of the present study represent the functional result of the various stimuli on mitochondria during the septic process in contrast to experiments using different markers of mitochondrial biogenesis where the relationship to functional outcome is hard to predict. We propose that a more severe septic insult or a more marked host response to the bacterial invasion leads to higher levels of cytokines and stimulants of mitochondrial biogenesis resulting in a more elevated respiratory capacity in non-survivors. Lending support to this is the recent study by Kellum *et al*. showing that 90-day mortality was higher in patients with severe sepsis that had the highest cytokine response when presenting at the emergency department [34].

In contrast to several other studies [35-37] we were unable to detect any functional inhibition of the respiratory complexes in the septic patients. These differences could be related to animal vs. human subjects, tissue specificity or different experimental conditions. In a high turnover cell type such as platelets it is also possible that an early initiation of the stimulatory process to increase respiration seen in the present study obscures an eventual early negative influence on mitochondrial respiration.

The limitations of the present study include the relatively small group sizes. Further, the generalizability of the present findings to other vital organ systems is at present not known.

## Conclusions

To our knowledge this is the first study of temporal changes in mitochondrial respiratory function in intact, viable and permeabilized platelets of septic patients. Platelets are an easy accessible source of viable mitochondria and proved to be well suited for repeated sampling and analysis of respiration. The present data indicate the presence of a soluble plasma factor in the initial stage of sepsis inducing uncoupling of platelet mitochondria leading to a decreased control ratio but no inhibition of respiratory complexes. The mitochondrial uncoupling was paralleled by a gradual and pronounced increase in mitochondrial respiratory capacity. This probably reflects a compensatory mitochondrial biogenic response to severe sepsis or septic shock, that was most pronounced in non-survivors, likely correlating to the severity of the septic insult.

## Key messages

• In the early phase of sepsis, intact platelets suspended in their own plasma (but not in other media) demonstrated an elevated basal non-phophorylating respiration (state 4) indicating a respiratory uncoupling mediated by a soluble factor in plasma.

• Multiple parameters of platelet mitochondrial respiratory capacity gradually and substantially increased during the first week of sepsis.

• Non-survivors at 90 days demonstrated more elevated respiratory capacities compared to survivors.

## Abbreviations

APACHE II: acute physiology and chronic health evaluation score; CI: complex I; CII: complex II; CLP: cecal ligation and puncture; CRP: C-reactive protein; Cyt *c*: cytochrome *c*; ETS: electron transfer system; FCCP: carbonyl cyanide p-(trifluoromethoxy) phenylhydrazone; MiR05: mitochondrial respiration media; MOF: multiple organ failure; mPT: mitochondrial permeability transition; mtDNA: mitochondrial DNA; NFR: nuclear respiratory factor; OXPHOS: oxidative phosphorylation; PBS: phosphate buffered saline; PCT: procalcitonin; PGC-1α: profilerator-activated receptor γ coactivator -1α; PRP: platelet rich plasma; ROS: reactive oxygen species; SAPS: simplified acute physiology score; SOFA: sequential organ failure assessment; SUIT: substrate: uncoupler: inhibitor titration; UCPs: uncoupling proteins.

## Competing interests

Erich Gnaiger is the founder of Oroboros Instruments, Austria and has developed the oxygraph used in the present study. The other authors declare no competing interests.

## Authors' contributions

FS, MH, EG and EE designed the study. FS, MH, EE, EG and HF interpreted the results. FS and SM collected data and samples and performed the experiments. FS drafted the manuscript. All authors read and approved the final manuscript.
